# Pharmacokinetics of fluralaner in dogs following a single oral or intravenous administration

**DOI:** 10.1186/1756-3305-7-85

**Published:** 2014-03-07

**Authors:** Susanne Kilp, Diana Ramirez, Mark J Allan, Rainer KA Roepke, Martin C Nuernberger

**Affiliations:** 1 MSD Animal Health Innovation GmbH, Zur Propstei, 55270 Schwabenheim, Germany; 2 Harlan Laboratories, Centro Industrial Santiga, c/Argenters, 6, 08130 Santa Perpètua de Mogoda Barcelona, Spain

**Keywords:** Fluralaner, Pharmacokinetics, Dog, Oral, Intravenous

## Abstract

**Background:**

Fluralaner is a novel systemic insecticide and acaricide. The purpose of these studies was to investigate the pharmacokinetic properties of fluralaner in Beagle dogs following single oral or intravenous (i.v.) administration.

**Methods:**

Following the oral administration of 12.5, 25 or 50 mg fluralaner/kg body weight (BW), formulated as chewable tablets or i.v. administration of 12.5 mg fluralaner/kg BW, formulated as i.v. solution to 24 Beagles, plasma samples were collected until 112 days after treatment. Plasma concentrations of fluralaner were measured using HPLC-MS/MS. Pharmacokinetic parameters were calculated by non-compartmental methods.

**Results:**

After oral administration, maximum plasma concentrations (C_max_) were reached within 1 day on average. Fluralaner was quantifiable in plasma for up to 112 days after single oral and i.v. treatment. The apparent half-life of fluralaner was 12–15 days and the mean residence time was 15–20 days. The apparent volume of distribution of fluralaner was 3.1 L/kg, and clearance was 0.14 L/kg/day.

**Conclusions:**

Fluralaner is readily absorbed after single-dose oral administration, and has a long elimination half-life, long mean residence time, relatively high apparent volume of distribution, and low clearance. These pharmacokinetic characteristics help to explain the prolonged activity of fluralaner against fleas and ticks on dogs after a single oral dose.

## Background

Fluralaner is a novel systemic insecticide and acaricide formulated as a chewable oral tablet that can be administered to dogs at 12 week intervals for effective persistent killing of a number of flea and tick species of dogs [1,2].

Fluralaner belongs to a new class of chemicals with an isoxazoline structure as an essential feature [2,3]. It has a molecular weight of 556.29, a log P_ow_ (octanol/water partition co-efficient) of 5.35 and is highly bound to plasma proteins.

Fluralaner is a potent inhibitor of ligand gated chloride channels (γ-aminobutyric acid (GABA)- and L-glutamate-gated chloride channels) in neurons with significant selectivity for arthropod neurons over mammalian neurons [2,3]. *In vivo* investigations have shown that after a single oral administration to dogs, fluralaner provides persistent flea and tick killing activity for 12 weeks [1]. Fluralaner is an innovative, highly effective, and long-lasting ectoparasite treatment for dogs.

## Methods

Chewable tablets containing 13.64% (w/w) fluralaner were formulated for oral administration to dogs. Test material for i.v. administration was formulated as solution with 2.5 mg fluralaner /mL in a Polyethylene glycol (PEG) 200 (90% v/v) vehicle containing 10% v/v water for injection.

Healthy male and female Beagle dogs were kept indoors in pens with sealed floors and individually housed until 5 or 6 weeks after fluralaner administration (oral or i.v.) to avoid potential cross contamination between animals. Thereafter, dogs were housed in groups of 3 of the same treatment group and sex. Room environment was monitored continuously, with a temperature of 15-21°C, relative humidity of 40-70%, 10–20 air changes per hour and a 12-hour fluorescent light/12-hour dark cycle. Dogs were fed once daily in the morning with a standard dog diet and had *ad libitum* access to water. On the day of oral fluralaner administration, the dogs received one-half of their daily food ration shortly before treatment and the remainder immediately after treatment, because administration of chewable tablets containing fluralaner with food increases its bioavailability [4].

To determine the rate and extent of systemic exposure after oral administration and oral dose proportionality, a parallel-group study was conducted with 3 treatment groups. Dogs received either 12.5, 25, or 50 mg (target dose) fluralaner/kg BW orally, with the mid dose (25 mg/kg BW) based on the minimum recommended treatment dose [1]. Additional pharmacokinetic parameters such as total body clearance and volume of distribution were determined in a separate study involving 6 Beagles administered 12.5 mg fluralaner/kg BW by slow i.v. infusion. All dose rates in the following sections are expressed in mg fluralaner per kg BW.

In both studies, dogs were randomized to treatment groups (3 dogs per sex per group), within sex, and blocked by body weight to ensure a balanced distribution. Both studies were compliant with the principles of Good Laboratory Practice [5]. The animal work was conducted in compliance with respective national legislation and approved by Animal Experimentation Ethics Committee at Harlan Laboratories S.A.

Individual oral doses were determined on the basis of individual body weights and the nominal content of fluralaner in the tablets. Dogs received whole tablets using either individual 112.5 mg, 250 mg or 500 mg fluralaner tablets, or a combination of tablets to achieve a dose close to the calculated target dose. Oral dosing was facilitated by placing the calculated target dose on the back of the dog’s tongue. Intravenous injection dose volumes were determined on the basis of individual body weights and were administered as a constant rate infusion over 5 minutes using an automatic injection system (KDS Model 200, KD Scientific Inc., Holliston, USA). The injection rate per hour was approximately 12 times the respective dose volume to ensure complete administration within 5 minutes.

Blood samples were collected from the jugular vein into sodium-citrate tubes before and at 2, 4, and 8 hours and 1, 2, 3, 4, 7, 14, 21, 28, 35, 42, 49, 56, 63, 70, 77, 84, 91, 98, 105, and 112 days after oral dosing and 15 min, 2, 4 and 8 hours, and 1, 2, 3, 4, 7, 14, 21, 28, 35, 49, 63, 77, 91, and 112 days after i.v. dosing. Plasma was harvested by centrifugation and stored frozen in sterile plastic vials until analysis. The dogs were closely observed for 1 hour after dosing and once daily thereafter.

Plasma samples were extracted by protein precipitation with acetonitrile and diluted with 0.1% formic acid. The resultant solution was analyzed quantitatively using automated solid phase extraction coupled to liquid chromatography with mass spectrometric detection (online SPE-HPLC-MS/MS). The linear range of the method for determination of fluralaner was 10.0 to 2500 ng/mL, with a lower limit of quantification (LLOQ) of 10.0 ng/mL. Pharmacokinetic parameters for fluralaner were calculated using non-compartmental methods with the validated software WinNonlin® Professional Version 5.3 (Pharsight Corporation, California, USA). The peak plasma concentration (C_max_) and time to peak concentration (t_max_) were observed values. The elimination half-life (t_1/2_) was calculated by linear regression using the slope of the terminal segment of the semilogarithmic plasma concentration versus time curve. The area under the concentration versus time curve (AUC) from time 0 to the last measurable concentration (AUC_(0→t)_) was calculated using the linear trapezoidal rule. The AUC from time 0 extrapolated to infinity (AUC_(0→∞)_) was determined as *AUC*_*(0→ t)*_ *+ C*_*t*_*/λ*_*z*_, where C_t_ is the plasma concentration at time t and λ_z_ is the first order rate constant associated with the terminal (log-linear) portion of the curve. The apparent volume of distribution (V_z_) after i.v. administration, based on the terminal phase was calculated as *Dose/λ*_*z*_ *× AUC*. Total body clearance (Cl) after i.v. administration was calculated as *Dose/AUC*. Bioavailability (F%) via the oral route was calculated using mean AUC_(0→∞)_ as *(AUC*_*(0→∞) oral*_*/AUC*_*(0→∞) iv*_*) × (Dose*_*iv*_*/Dose*_*oral*_*) × 100.* Mean residence time (MRT) extrapolated to infinity was calculated as the ratio of *AUMC/AUC*; where AUMC is the area under the first moment curve. Dose proportionality was tested for exposure parameters C_max_ and AUC_(0→t)._ For this purpose dose-normalized (nominal dose) values were analyzed using an appropriate analysis of variance (ANOVA) regression model. The tests were two-sided at the 0.05 level of significance. All data are expressed as arithmetic mean ± SD unless otherwise stated.

## Results and discussion

Each of three groups of six dogs (age 1–2 years; weight 7.8-11.4 kg) was administered fluralaner orally at one of three target doses (12.5, 25, or 50 mg/kg). The doses were achieved using whole tablets, and therefore each dog received a dose around the target dose. This was a range of 12.0–14.6 mg/kg for a target dose of 12.5 mg/kg, 25.0–28.8 mg/kg for a target dose of 25 mg/kg, and 42.2–53.2 mg/kg for a target dose of 50 mg/kg.

No dog showed any clinical finding or adverse event after oral fluralaner administration.

**Figure 1:**
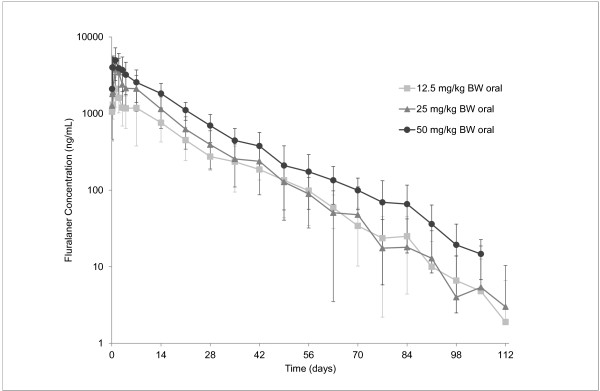
**Plasma concentrations of fluralaner (Mean ± Standard Deviation) in dogs following single oral administration.** Values below LLOQ (10 ng/mL) were set to zero for calculation of means.

Plasma concentrations of fluralaner reached C_max_ within 1 day after administration on average, and progressively declined over time, with some lower secondary peaks, which may indicate redistribution or recirculation e.g. enterohepatic re-circulation. There was a dose-dependent increase in mean peak plasma concentrations. The terminal portion of the plasma concentration versus time curves followed an almost parallel course; in the lower two dose groups at the same concentration with high inter-individual variability, and in the high dose group at a higher concentration with lower inter-individual variability. Fluralaner could be quantified in plasma (> 10 ng/mL) for up to 112 days after treatment, demonstrating a long systemic persistence (Figure 1).

**Figure 2:**
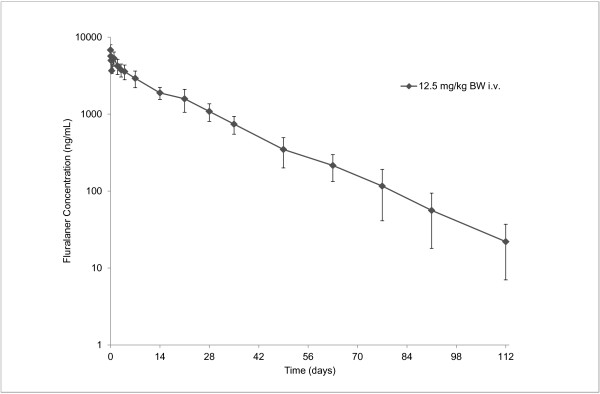
**Plasma concentrations of fluralaner (Mean ± Standard Deviation) in dogs following single i.v. administration.** Values below LLOQ (10 ng/mL) were set to zero for calculation of means.

In the i.v. (12.5 mg/kg) group very slow elimination of fluralaner was observed after the 5-minute infusion. In all dogs, plasma concentrations of fluralaner declined as a function of time, with some secondary peaks, which may indicate redistribution or recirculation e.g. enterohepatic re-circulation. Fluralaner could be quantified in plasma for up to 112 days post administration, demonstrating a long systemic persistence after i.v. administration to dogs (Figure 2).

The non-compartmental pharmacokinetic parameters calculated from the concentration–time data of fluralaner are shown in Table 1.

**Table 1:** Plasma pharmacokinetic parameters of fluralaner in dogs after either single oral or single i.v. administration

**Parameters**	**Oral 12.5 mg/kg n = 6**	**Oral 25.0 mg/kg n = 6**	**Oral 50.0 mg/kg n = 6**	**Intravenous 12.5 mg/kg n = 6**
C_max_ (ng/mL)	2144 ± 860	3948 ± 1734	5419 ± 2086	7109 ± 908
t_max_^a^ (day)	1 (range 0.08–2)	1 (range 1–2)	1 (range 0.17–3)	n/a
AUC_(0-d112)_ (day* ng/mL)	29665 ± 13858	46115 ± 18932	70171 ± 26412	87198 ± 11835
AUC_(0→∞)_ (day*ng/mL)	29922 ± 13808	46416 ± 18929	70531 ± 26529	87779 ± 12004
t_1/2_ (day)	13 ± 1	12 ± 3	14 ± 1	15 ± 2
MRT (day)	19 ± 2	15 ± 4	17 ± 3	20 ± 3
Cl (L/kg/day)	n/a	n/a	n/a	0.14 ± 0.02
V_z_ (L/kg)	n/a	n/a	n/a	3.1 ± 0.5

^a^Median, other values are mean ± standard deviation.

n/a - not applicable.

The mean total plasma clearance of fluralaner was 0.14 L/kg/day and the mean apparent volume of distribution of fluralaner was 3.1 L/kg following i.v. infusion.

The mean half-life (15 days) and mean residence time (20 days) values suggest a slow elimination process following i.v. infusion. These parameters were similar to values determined after oral administration at different dose rates, indicating that the elimination kinetic appears to be independent of dose and administration route.

As only unbound drugs in the vascular system are available to clearing organs for elimination, apparent volume of distribution and clearance are determinants of the terminal half-life [7-9]. Taking into account the total body water volume (approximately 0.6 L/kg) of a dog [6], fluralaner has a relatively high apparent distribution (V_z_ = 3.1 L/kg) into tissues following i.v. infusion, despite its high level of binding to plasma proteins.

For fluralaner, it is assumed that the main route of elimination is likely hepatic, because the high plasma protein binding suggests that elimination via renal filtration is minor; therefore, plasma clearance can be assumed to be equivalent to hepatic clearance. Considering a physiological hepatic blood flow in the dog of approximately 44.5 L/kg/day [6,7] and hepatic clearance of fluralaner of 0.14 L/kg/day, the hepatic extraction ratio is estimated to be low (0.3%). The low clearance, together with the relatively high distribution to tissues, may explain the long-lasting systemic availability of fluralaner in the dog.

The bioavailability of the oral formulation is slightly higher at lower oral doses (34 ± 16%, 26 ± 11%, and 20 ± 8%, for 12.5, 25 and 50 mg/kg, respectively).

However, there was no statistically significant differences between dose groups in dose-normalized exposure parameters AUC_(0→t)_ and C_max_ (ANOVA, *p* = 0.165 for AUC_(0→t)_ and *p* = 0.206 for C_max_), indicating no evidence against the null hypothesis of fluralaner dose proportionality over the dose range 12.5 mg/kg to 50 mg/kg.

## Conclusions

Fluralaner is readily absorbed after single-dose oral administration, and has a long elimination half-life, long mean residence time, relatively high apparent volume of distribution, and low clearance. These pharmacokinetic characteristics help to explain the prolonged activity of fluralaner against fleas and ticks on dogs after a single oral dose.

### Compliance statement

This study was conducted in Spain after obtaining the authorization of the relevant regulatory authorities.

## Competing interests

All authors, except DR, are employees of Merck/MSD Animal Health.

## Authors’ contributions

SK, DR, MJA, RKAR & MCN authored the study design and protocol, monitored the study and interpreted the results. SK drafted the manuscript and all authors revised and approved the final version.

## References

[B1] Rohdich N Roepke RKA Zschiesche E A randomized, blinded, controlled and multi-centered field study comparing the efficacy and safety of Bravecto^TM^ (fluralaner) against Frontline^TM^ (fipronil) in flea- and tick-infested dogs Parasit Vectors 2014 7 83 10.1186/1756-3305-7-83 24593931 PMC3975895

[B2] Gassel M Wolf C Noack S Williams H Ilg T The novel isoxazoline ectoparasiticide fluralaner: selective inhibition of arthropod γ-aminobutyric acid- and L-glutamate-gated chloride channels and insecticidal/acaricidal activity Insect Biochem Mol Biol 2014 45 111 124 24365472 10.1016/j.ibmb.2013.11.009

[B3] Ozoe Y Asahi M Ozoe F Nakahira K Mita T The antiparasitic isoxazoline A1443 is a potent blocker of insect ligand-gated chloride channels Biochem Biophys Res Commun 2010 391 744 749 10.1016/j.bbrc.2009.11.131 19944072

[B4] Walther FM Allan MJ Roepke RKA Nuernberger MC The effect of food on the pharmacokinetics of oral fluralaner in dogs Parasit Vectors 2014 7 84 10.1186/1756-3305-7-84 24598049 PMC3975707

[B5] OECD Series on Principles of Good Laboratory Practice and Compliance Monitoring No.1. OECD Principles of Good Laboratory Practice (as revised in 1997) 1998 Paris: OECD Environmental Health and Safety Publications, ENV/MC/CHEM(98)17

[B6] Davies B Morris T Physiological parameters in laboratory animals and humans Pharm Res 1993 10 7 1093 1095 10.1023/A:1018943613122 8378254

[B7] Toutain PL Bousquet-Mélou A Clearance J Vet Pharmacol Ther 2004 27 415 425 10.1111/j.1365-2885.2004.00605.x 15601437

[B8] Toutain PL Bousquet-Mélou A Plasma terminal half life J Vet Pharmacol Ther 2004 27 427 439 10.1111/j.1365-2885.2004.00600.x 15601438

[B9] Toutain PL Bousquet-Mélou A Volumes of distribution J Vet Pharmacol Ther 2004 27 441 453 10.1111/j.1365-2885.2004.00602.x 15601439

